# How and Why the Circadian Clock Regulates Proliferation of Adult Epithelial Stem Cells

**DOI:** 10.1093/stmcls/sxad013

**Published:** 2023-02-06

**Authors:** Bogi Andersen, Junyan Duan, Satya Swaroop Karri

**Affiliations:** Division of Endocrinology, Department of Medicine, School of Medicine, University of California, Irvine, CA, USA; Department of Biological Chemistry, School of Medicine, University of California, Irvine, CA, USA; Institute for Genomics and Bioinformatics, University of California, Irvine, CA, USA; Center for Complex Biological Systems, University of California, Irvine, CA, USA; The NSF-Simons Center for Multiscale Cell Fate Research, University of California Irvine, Irvine, CA, USA; Center for Complex Biological Systems, University of California, Irvine, CA, USA; The NSF-Simons Center for Multiscale Cell Fate Research, University of California Irvine, Irvine, CA, USA; Department of Biological Chemistry, School of Medicine, University of California, Irvine, CA, USA

**Keywords:** adult stem cells, aging, cell cycle, epidermis, stem cell cycle

## Abstract

First described in the early 20th century, diurnal oscillations in stem cell proliferation exist in multiple internal epithelia, including in the gastrointestinal tract, and in the epidermis. In the mouse epidermis, 3- to 4-fold more stem cells are in S-phase during the night than during the day. More recent work showed that an intact circadian clock intrinsic to keratinocytes is required for these oscillations in epidermal stem cell proliferation. The circadian clock also regulates DNA excision repair and DNA damage in epidermal stem cells in response to ultraviolet B radiation. During skin inflammation, epidermal stem cell proliferation is increased and diurnal oscillations are suspended. Here we discuss possible reasons for the evolution of this stem cell phenomenon. We argue that the circadian clock coordinates intermediary metabolism and the cell cycle in epidermal stem cells to minimize the accumulation of DNA damage from metabolism-generated reactive oxygen species. Circadian disruption, common in modern society, leads to asynchrony between metabolism and the cell cycle, and we speculate this will lead to oxidative DNA damage, dysfunction of epidermal stem cells, and skin aging.

## Graphical Abstract

**Graphical Abstract:**
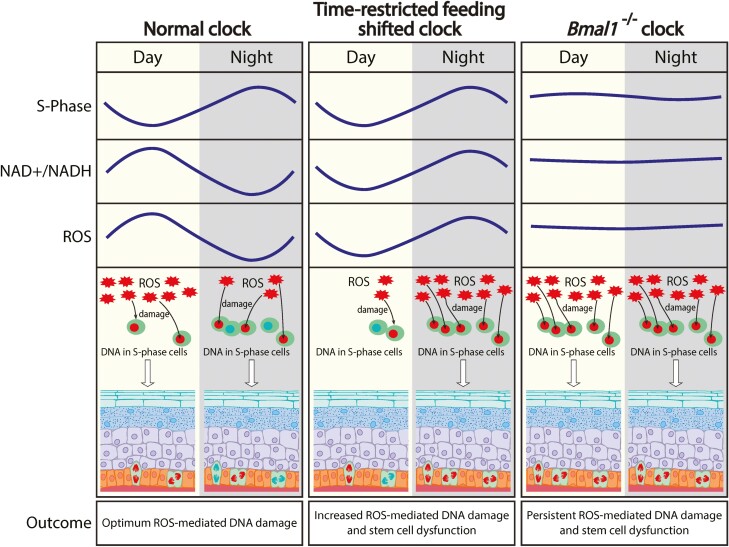

**Significance Statement**

This Concise Review describes how the circadian clock regulates proliferation of stem cells. Disruption of the circadian clock, which is common in modern society, may influence the function of adult stem cells, contributing to diseases such as aging and impaired regeneration.

Work over the last 15 years has revealed a role for the circadian clock in regulating properties and function of adult stem cells,^1^ including hematopoietic stem cells,^2^ intestinal stem cells,^3^ and various populations of skin stem cells.^4,5^ This Concise Review focuses on the role of the circadian clock in regulating the proliferation of interfollicular epidermal (IFE) stem cells, an important model for understanding how and why the circadian clock regulates adult epithelial stem cells.

## Adult Epithelial Stem Cells

**Figure 1.**
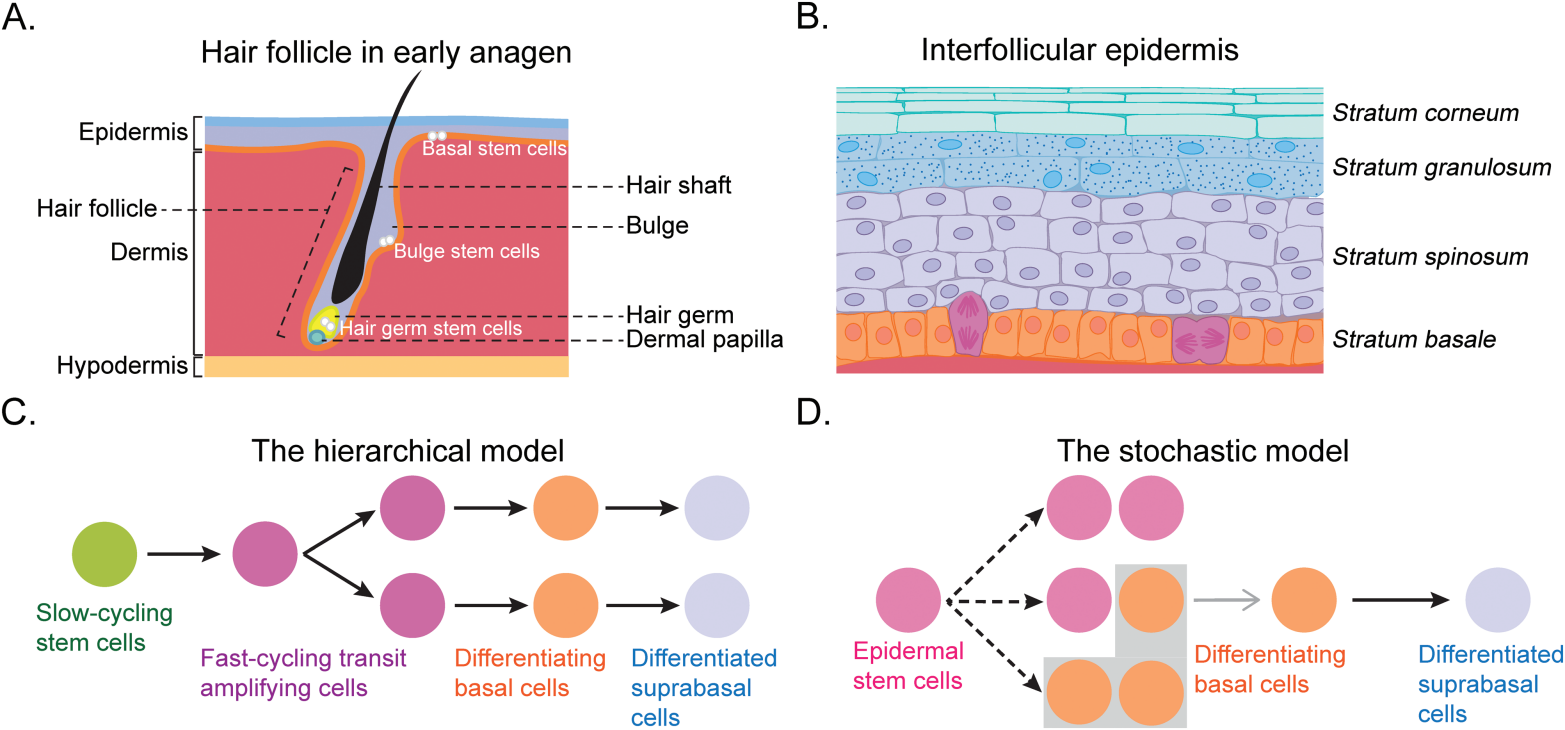
Skin stem cells. **A**. Organization of hair follicles. The hair follicles represent invaginations from the epidermis. The bulge region contains slowly cycling stem cells that are activated to replenish cells for the growing hair follicle. The actively proliferating hair germ, thought to be composed of stem and progenitor cells, expands at the beginning of hair growth (anagen) and supplies most of the cells for the growing hair follicle. The dermal papilla, a mesenchymal component of the hair follicle, signals to the overlying hair germ. **B**. A schematic of the interfollicular epidermis (IFE). The IFE represents the outermost layer of the skin, overlaying the dermis, which overlays the hypodermis (dermal fat) and the subcutaneous layer. It is composed of the stratum basale, which contains the stem cells, and 2 layers of progressively differentiating epidermal cells, the stratum spinosum and stratum granulosum. The stratum corneum represents dead anucleated cells that eventually are shed from the surface. The image represents a stylized version of the human epidermis. The adult mouse epidermis is much thinner. **C**. The hierarchical model for IFE stem cells. This model posits there are rare slow-cycling stem cells that give rise to rapidly proliferating transit amplifying progenitor cells. **D**. The stochastic model for IFE stem cells. This model posits that cells of the basal layer are equivalent and that they can divide either symmetrically (to give rise to 2 stem cells or 2 differentiating cells) or asymmetrically (to give rise to a stem cell and a differentiating cell).

The defining features of adult epithelial stem cells are their abilities to self-renew, to give rise to differentiated cells of the epithelium, and to maintain the stem cell niche.^6^ In addition to maintaining tissue homeostasis, epithelial stem cells are activated to coordinate tissue repair after injury and wounding.^7^ Skin contains a number of different types of stem cells.^8^ These include quiescent hair follicle bulge stem cells, which are intermittently activated to supply cells for the growing hair follicles, as well as actively proliferating Lgr5-positive stem- and progenitor cells of the secondary hair germ (Fig. 1A).

Contrasting the intermittently proliferating stem cells of the hair follicle, the basal cell layer of the IFE contains constantly proliferating stem cells that match corneocyte loss from the top of the epidermis to maintain homeostasis of the IFE (Fig. 1B). The nature of IFE stem cells remains controversial. According to a hierarchical model (Fig. 1C), the IFE basal cell layer contains rare slow-cycling stem cells that give rise to fast-cycling transit amplifying cells that lack stem cell features but represent most proliferating cells of the IFE.^9^ A variant of this model posits that the basal cell layer contains 2 types of slow-cycling stem cells, which can be identified based on marker expression; this model may explain how the scale and interscale regions of the mouse tail skin form.^10^ Contrasting these hierarchical models, other data suggest that cells of the basal cell layer are equivalent and that stochastic choice (Fig. 1D) determines which cells proliferate and which cells differentiate.^11,12^ Consistent with this model, in vivo imaging shows that random differentiation of IFE stem cells triggers cell division of adjacent stem cells.^13^

Although single-cell RNA-sequencing experiments have identified distinct basal cell layer clusters in the IFE of mice^14-16^ and humans,^17^ these clusters do not match well with rare populations of stem cells. In fact, the single-cell RNA-sequencing experiments point to gradual differences and a continuous differentiation trajectory of the IFE basal cells,^16^ which is consistent with evidence pointing to the flexibility of stem cells.^18^ Here, we use the term IFE stem cells synonymously with basal cell layer IFE cells.

## The Circadian Clock

**Figure 2.**
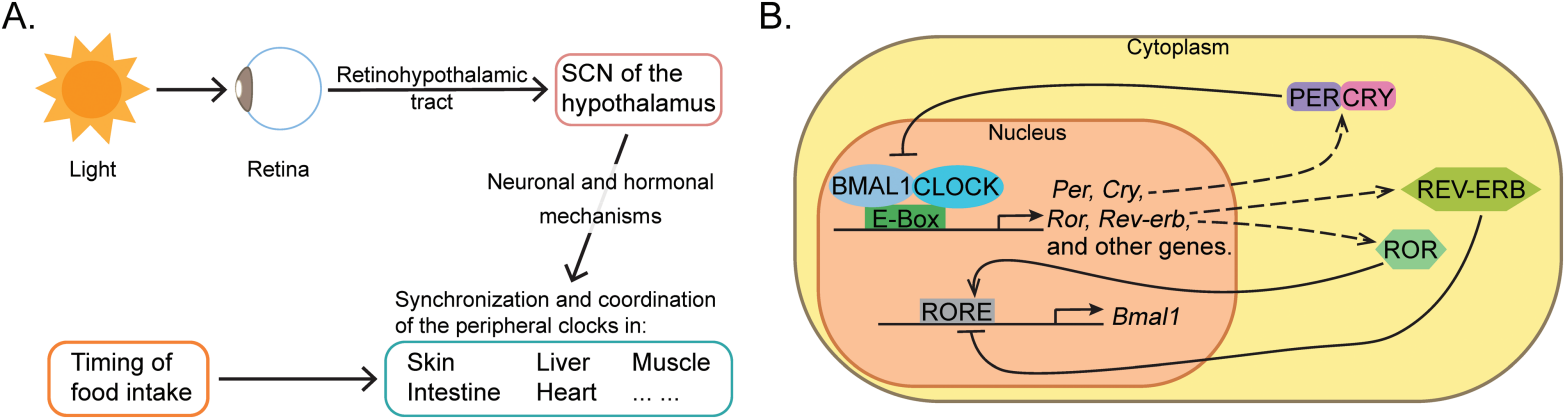
The circadian clock. **A**. The physiology of the circadian clock. The autonomous, central clock resides in the suprachiasmatic nucleus (SCN) of the hypothalamus. Its timing is set by light signals conveyed from the retina via the retinohypothalamic track. The SCN synchronizes and coordinates clocks in other cells, including in skin, via hormonal and direct neuronal mechanisms. Other signals such as food intake can set the timing of clocks in peripheral tissues without altering the SCN clock. **B**. The molecular mechanisms of the clock. The transcription factors BMAL1 and CLOCK bind to E-boxes and drive the expression of multiple genes, including their own inhibitors, PER and CRY. The nuclear receptors ROR and REV-ERB form an auxiliary loop where they, respectively, activate and inhibit the expression of *Bmal1*. RORE, DNA-binding element for RORs.

All organisms harbor a circadian clock, a self-sustaining mechanism that adjusts the body’s physiology to better handle environmental and intrinsic changes stemming from the 24-h rotation of the Earth.^19^ The mammalian circadian clock was initially thought to be located only in neurons of the suprachiasmatic nucleus (SCN), but later work revealed that all or nearly all cells of the body contain an active circadian clock. The SCN clock, which is set by light signals from the retina, synchronizes clocks in peripheral tissues through hormonal and direct neuronal mechanisms (Fig. 2A). As light entrains the SCN clock, it is referred to as a zeitgeber. In experiments where 12-h light and dark cycles are used, Zeitgeber Time (ZT) 0 refers to time of lights on and ZT12 to time of lights off.

Clocks in peripheral organs, including skin, are also subject to regulation by time of feeding.^20^ Although ablation of the SCN leads to circadian arrhythmia in skin^21,22^ and circadian asynchrony between organs,^23^ it is not fully understood to which extent circadian rhythms in peripheral tissues can be maintained in the absence of the SCN clock.^23,24^ Studies in skin showed, surprisingly, that mice harboring *Bmal1* only in the epidermis could maintain diurnal rhythms if daily day-night cycles were maintained.^25^

At a molecular level, the clock is created by a negative feed-back loop where heterodimeric bHLH-PAS transcription factors, BMAL1 and CLOCK, drive the expression of their own inhibitors, PER (PER1, 2, and 3) and CRY (CRY1 and 2) (Fig. 2B).^26^ BMAL1 and CLOCK also drive expression of nuclear receptors ROR (RORa, b, and c) and REV-ERB (NR1D1, NR1D2), which activate and inhibit, respectively, expression of BMAL1. This core transcriptional network also creates diurnal oscillations in the expression of approximately 10% of the transcriptome in each tissue, often affecting genes that participate in the characteristic physiological function of each organ.^27^

Epidemiological studies have linked shift work and circadian disruption to a number of diseases that involve epithelial stem cells, including skin diseases and cancer.^28^ Systematic analysis across 32 human cancers revealed widespread clock gene alterations and disrupted circadian rhythms that correlated with oncogenic pathways and survival.^29^ Consistent with these findings, studies in mice have shown that jet lag paradigms or genetic circadian disruption accelerate lung^30^ and intestinal^31,32^ tumorigenesis.

## How Does the Circadian Clock Regulate Epidermal Stem Cells?

Although the circadian clock has important roles in adult epithelial stem cells, there is little evidence for its importance in epithelial stem cells during embryonic development. Mice with mutations in core clock genes are born with normal skin and gastrointestinal system and the circadian clock does not seem to be fully functional in the skin until postnatally.^33^

### Regulation of Hair Follicle Stem Cell Proliferation

 In the hair follicle bulge, the clock contributes to the heterogeneity of quiescent stem cells by regulating the sensitivity to inhibitory (TGFβ) and activating (Wnt) signals.^34^ Thus, for the slow-cycling hair follicle stem cells, the circadian clock may help select cells for activation during hair growth. MPZL3, a nuclear-encoded, mitochondrially localized, immunoglobulin-like V-type protein, has also been proposed as a link between the circadian clock and hair follicle cycling.^35^

As hair growth is initiated, the circadian clock genes are expressed to high levels in the secondary hair germ of the early anagen follicle, which is composed of actively cycling stem- and progenitor cells.^36^ In mice deleted for *Bmal1* globally, the secondary hair germ cells downregulate REV-ERBα, which leads to upregulation of the G1 cell cycle inhibitor p21; this may arrest the secondary hair germ cells at the G1-S cell cycle stage and delay initiation of hair growth by several days.^36,37^ Loss of PTEN, a major regulator of the PI3K-Akt pathway, leads to upregulation of BMAL1 and increased number of hair follicle stem cells, which expand in a BMAL1-dependent manner.^38^

Later studies showed that when *Bmal1* was specifically deleted in keratinocytes, no delay in hair growth was observed,^39^ suggesting that the clock modulates hair growth through non-keratinocyte skin cells or through systemic effects.^40^ Another study pointing to indirect regulation of hair follicle stem cells showed that light stimulus to the eyes activates retinal ganglion cells, which project to the SCN. The SCN then activates sympathetic nerves projecting to skin where they promote the proliferation of hair follicle stem cells via hedgehog signaling.^41^

### Regulation of IFE Stem Cell Proliferation

**Figure 3.**
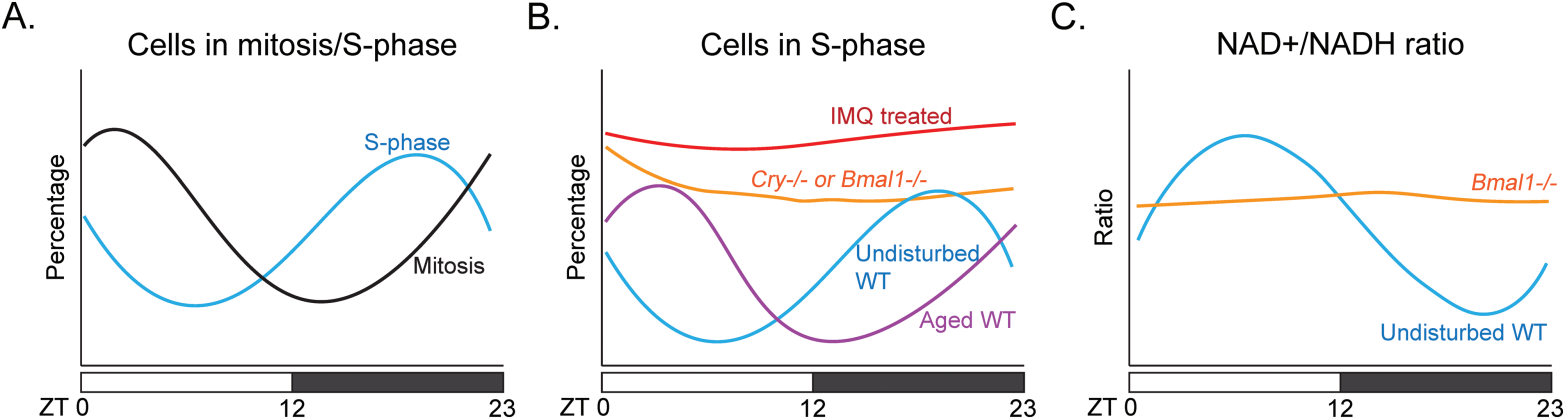
Diurnal proliferation and metabolism in interfollicular epidermal stem cells. **A**. Oscillation in S-and M-phase. IFE stem cells show a peak in S-phase in late night and a peak in M-phase approximately 6 h later, in the early day. **B**. Disruption of diurnal oscillations in IFE stem cell proliferation. Mutations in core clock genes lead to a constant cell proliferation at a level that is high. In skin inflammation, such as induced by the TLR7 agonist Imiquamod (IMQ), IFE stem cell proliferation is high and invariant over the day. Aging shifts the peak in S-phase by several hours. **C**. Diurnal variation in NAD/NADH ratio. The diurnal variation in NAD/NADH ratio is obliterated when *Bmal1* is mutated.

Studies going back to the early 20th century showed diurnal variation in epithelial cell mitotic activity, including in the epidermis.^42,43^ Later studies characterized the daily cycling of epithelial stem cells in multiple epithelial organs including the gut^44^ and human skin.^45^ In mice, the proliferation of IFE stem cells oscillates over the day with maximum fraction of stem cells in S-phase at night between 11 PM and 3 AM (ZT17-21) and minimum fraction of cells in S-phase during the day between 11 AM and 3 PM (ZT5-9) (Fig. 3A).^39^ Maximum and minimum fractions of IFE stem cells in mitosis are observed approximately 6 h after, respectively, the peak and trough in S-phase^39,46^ (Fig. 3A), indicating that the whole cell division cycle of IFE stem cells is diurnal.

It was not until the early 21st century that experiments with mouse mutants showed that the intact circadian clock is required for these daily oscillations in IFE stem cell proliferation.^39,47^ Circadian S-phase in IFE stem cells is obliterated with deletion of both *Cry* genes^47^ or *Bmal1*, either globally or specifically in keratinocytes^39^ (Fig. 3B), indicating that the clock intrinsic to epidermal cells is critical for this function. In *Bmal1*-mutated keratinocytes, the IFE stem cells proliferate at a constant high rate,^39^ indicating that the physiological role of the clock is to suppress DNA synthesis in IFE stem cells during the day. This suppressive function of the circadian clock in IFE stem cells contrasts with its activation of cell proliferation in the secondary hair germ, highlighting the versatility of the circadian clock.

The upper parts of hair follicles renew in a continuous manner similar to the IFE, and the circadian clock affects the proliferation of these cells in a manner similar to the IFE.^39^ In the matrix of actively growing hair follicles, which have high mitotic rates, the diurnal rhythms in cell proliferation are dampened^39^ as described for other highly proliferative epithelia, including the seminiferous tubules.^43^ The dampened diurnal variation in cell proliferation correlates with dampened amplitude of circadian clock genes in the highly proliferative cell compartments of hair follicles.^36,39^ Such dampened or suspended circadian rhythms have been described in other highly proliferative tissues, such as seminiferous tubules^48^ and thymus.^49^ Yet, proliferating cells of the hair matrix show diurnal sensitivity to gamma radiation-induced cell death that depends on a functional circadian clock.^50^

Although a functional circadian clock within epidermal cells is required for these daily cycles in IFE stem cell proliferation, the phase of the clock may not be a critical regulator of the phase of the cell cycle oscillations. Thus, when feeding is restricted to the day, the phase of the skin circadian clock is shifted by several hours as is the phase of many diurnally expressed cell cycle genes. But yet the phase of the S-phase cycles is unchanged.^20^ Restricted feeding, however, does decrease the overall proliferation rate of IFE stem cells,^20^ perhaps related to lower body weight under restricted feeding conditions. Other studies that restricted calories rather than the time of food intake showed IFE thickening and an increased number of IFE stem cells based on CD29 cell sorting.^51^

Under conditions of skin injury and inflammation, the proliferative proportion of IFE stem cells is increased and becomes invariant over the day^52^ (Fig. 3B). Apparently, when increased stem cell proliferation is needed in acute skin repair, the clock suppression of the cell cycle is suspended in IFE stem cells. Circadian rhythms are also commonly lost in epithelial cancers, including in human colorectal carcinoma.^31^ Furthermore, circadian clock genes are abnormally expressed in hyperproliferative psoriasis skin lesions.^52^

Although dampened circadian oscillations in the SCN have been postulated to contribute to aging,^53^ IFE stem cells maintain robust circadian rhythms into old age.^54^ But the peak in S-phase in aging IFE stem cells is delayed by several hours (Fig. 3B), and the circadian transcriptome switches to programs involved in cellular stress, such as DNA damage—a change that can be counteracted with calorie restriction.^54^

### Mechanisms Underlying Clock-Regulation of IFE Stem Cell Proliferation Oscillations

The mechanisms underlying circadian clock-regulation of oscillations in IFE stem cell proliferation remain unclear. Several studies point to the possibility of intrinsic mechanisms whereby circadian clock transcription factors directly control the expression of cell cycle regulators, as posited for the role of BMAL1 in the secondary hair germ where the circadian clock promotes stem cell proliferation.^36^

A study on the link between the circadian clock and cell proliferation in liver regeneration showed that the clock is required for expression of WEE1, a kinase involved in the G2/M transition and cell proliferation.^55^ This foundational study showed that the molecular clock directly regulates the expression of cell cycle proteins. *Wee1* is one of the most consistent circadian clock-regulated genes across tissues, including in the skin. But its role in clock-controlled IFE stem cell oscillations of proliferation has not been tested. The transcription factor KLF9 was proposed as a regulator of human epidermal stem cell proliferation.^56^ Its diurnal expression, however, is not antiphasic in mouse epidermis whereas S-phase is antiphasic to human epidermal stem cell S-phase. Therefore, to have a conserved role in regulating oscillations in IFE stem cells in mammals, KLF9 would have to have fundamentally different cell cycle target genes in mouse and human IFE stem cells.

In vitro studies have suggested that the clock and the cell cycle are interlocked mechanisms regulating each other intrinsically.^57,58^ Although these studies point to intrinsic mechanisms tightly coupling the circadian clock to the cell cycle, this model does not describe the in vivo situation for stem cells in either the epidermis or the gut where S-phase cells are found throughout the day, albeit in different proportions. The intrinsic model is also undercut by studies of daytime-restricted feeding which shifts the phase of the clock and the expression of many cell cycle regulators in skin without altering the timing of S-phase in IFE stem cells.^20^

Studies in other epithelia have pointed to external cell signaling being important for regulating diurnal cell divisions in stem and progenitor cells. In murine intestinal organoids grown in vitro, Paneth cells secrete WNT diurnally, which in turn controls the cell division of intestinal stem and progenitor cells in a circadian manner.^59^ The possibility that circadian clock-controlled signals external to IFE stem cells control their diurnal oscillations in proliferation remains unexplored. But if external signals are responsible, those must come from the epithelial cells because selective mutation of *Bmal1* within keratinocytes is sufficient to obliterate oscillations in IFE stem cell S-phase.^39^

### Regulation of the Response to UVB-Induced DNA Damage in the IFE

The circadian clock also regulates the ultraviolet (UV) response and UVB-induced DNA damage in IFE stem cells. When UVB is applied to mouse skin at night, there is a greater accumulation of cyclobutane pyrimidine dimers, 6-4 photoproducts, and double-stranded DNA breaks in IFE stem cells than when UVB is applied during the day.^39,47^ This diurnal variation is controlled by the circadian clock as deletion of both *Cry* genes or *Bmal1* obliterates the day-night differences in UVB-induced DNA damage.^39,47^

The enhanced sensitivity to UVB-induced DNA damage at night may link to the circadian clock-regulation of IFE stem cell proliferation because at night most cells are in S-phase,^39^ a cell cycle stage sensitive to UVB-induced DNA damage. In addition, the clock controls the expression of XPA, a rate-limiting enzyme in DNA excision repair, which is lowest during the night, indicating that DNA excision repair is less efficient at night.^47,60^ Daytime-restricted feeding decreases the expression of *Xpa* and reverses the daily rhythms in sensitivity to UVB-induced DNA damage.^20^ One of the consequences of this diurnal variation in UVB-induced DNA damage is that more skin tumors form in mice when UVB is applied at night than during the day.^47^

## Why Does the Circadian Clock Regulate Proliferation of IFE Stem Cells?

Why mammals have evolved diurnal variation in epithelial stem cell proliferation remains an area of active research. It is hard to appreciate how diurnal variation in stem cell proliferation during homeostasis would be beneficial for the formation of epithelial barriers, which are formed over the course of several days during differentiation. Here, we review 3 of the hypotheses that have been proposed to explain the circadian clock-controlled oscillations in epidermal stem cell proliferation.

### Adapting to Diurnal Rhythms in Solar Exposure

Studies in simpler organisms, which out in nature are fully exposed to solar radiation during the day, suggest that the clock has evolved to better deal with solar-induced DNA damage.^61^ Consistent with this idea, the DNA repair system is more active during the day than at night in such organisms. Because skin among other mammalian organs is uniquely exposed to UV radiation, this possibility deserves consideration for the mammalian skin clock. In particular, as previously discussed, mouse IFE stem cells have the lowest fraction of cells in S-phase and most active DNA excision repair during the day, which would fit with the solar exposure model.^39,47^

Yet, there are several reasons why adaptation to solar exposure is an unlikely explanation for the diurnal rhythms in mammalian epithelial stem cell DNA replication. First, in the wild, mice and other rodents are well protected from sunlight by their fur, and being nocturnal, they stay out of the sunlight during the day. Second, the diurnal rhythms in DNA replication and repair are not limited to epidermal stem cells but are also found in other epithelia that are not exposed to UV. Third, the diurnal cell division cycles in the epidermis of nocturnal rodents^39,46^ and diurnal humans^45,62^ are antiphasic. So, while mice may be better protected during the day, clock-regulation of IFE stem cells may make humans more sensitive to UVB-induced DNA damage during the day than the night.

### Adapting to Other Harmful Environmental Stressors

The epidermis is a barrier protecting us from harmful environmental effects. In addition to protection against solar radiation, the epidermis wards off pathogenic microorganisms, allergens, toxic pollutants, and physical injury. Upon injury, the regenerative response features multiple cell types, including hair follicle and IFE stem cells, fibroblasts, and immune cells, all of which are activated with increased proliferation and migration toward the injury site. Multiple aspects of the skin immune and injury response are under the influence of the circadian clock.^28,33^

Skin wound healing is impaired in animals with circadian disruption^63,64^ and in mice lacking BMAL1,^65^ possibly because of increased oxidative stress.^66^ The interferon response to a TLR7 agonist is highly diurnal and regulated by BMAL1 in the skin.^52^ Epidermal keratinocytes produce the chemokine CXCL14 in a circadian pattern, which suppresses skin bacterial proliferation in a diurnal manner through the regulation of the innate immune system.^67^ The migration of dermal dendritic cells into lymphatic vessels is under the control of the chemokine CCL21 and adhesion molecules, which in turn are directly regulated by the circadian clock.^68^

The likelihood of encountering harmful external stressors is greater during the active phase than during sleep^69^ and studies have shown that wounds incurred during the active phase heal faster than wounds incurred during the rest phase.^70^ It is, therefore, possible that an increased proportion of IFE stem cells in S-phase during the active phase has evolved to facilitate repair and regeneration in response to wounds and other cellular stressors. During homeostasis, the daily oscillations could be important as constant high activity of stress response pathways, immunity, and DNA synthesis, could increase the propensity to diseases such as autoimmunity and cancer.

### Adapting to Endogenous Diurnal Rhythms in Metabolism

Another hypothesis is that the daily rhythms in IFE stem cell proliferation have evolved to adapt to the internal environment of the body. This concept is derived in part from experiments in budding yeast showing that metabolic cycles are temporally compartmentalized and synchronized with other cellular functions.^71,72^ In mammals, the metabolic environment of day and night varies drastically, in part due to alterations in food intake and physical activity.

Such a model is also more consistent with the antiphasic relationship between the behavior of IFE stem cells in nocturnal and diurnal animals than the solar exposure model. In particular, the pattern of DNA synthesis and repair in IFE stem cells could decrease accumulating DNA damage from oxidative metabolism. Reactive oxygen species (ROS) are a normal byproduct of mitochondrial respiration^73^ and over an organism’s lifetime may lead to accumulation of cellular damage, including DNA mutations. The reliance of cancer cells and other highly proliferative cells on glycolysis may be to minimize cell-damaging oxidative stress.^74,75^

As S-phase is a cell cycle phase that is sensitive to ROS-mediated damage, there would be an advantage to having the fewest cells in S-phase during the time of maximum oxidative stress. Consistent with this model, the expression of OXPHOS genes and skin levels of reactive oxygen species (ROS) are antiphasic to the peak in IFE stem cell S-phase.^39^ Furthermore, the expression of many OXPHOS genes in skin is BMAL1-regulated, and in *Bmal1*-mutant mice, ROS levels rise and become invariant over the day.^39^

This idea has been studied in mouse skin using fluorescence lifetime imaging microscopy to measure the NADH/NAD+ redox ratio, a reflection of the relative ratio of glycolysis and OXPHOS in IFE stem cells.^76^ These experiments showed that IFE stem cells favor glycolysis during the night when the proportion of S-phase cells is highest and OXPHOS during the day when the proportion of S-phase cells is lowest. These daily fluctuations in the NADH/NAD+ redox ratio depend on BMAL1 (Fig. 3C). Furthermore, the heterogeneity in this ratio correlates with heterogeneity in circadian phase as measured in vivo in IFE stem cells expressing the fluorescent VENUS protein from the *Per1* promoter. In these studies, NADH/NAD+ ratio reflected the whole cell, but the majority of the signal came from mitochondria. A caveat is that lipid and carbohydrate metabolism may differentially affect the cytoplasmic and mitochondrial NADH levels.

Given that time of feeding alters the oxidative metabolic state of skin without altering the phase of cell division as measured by S-phase, it is likely that abnormal feeding times may lead to asynchrony between oxidative metabolism and DNA synthesis in IFE stem cells.^20^ Over the lifetime of an organism, this asynchrony could contribute to increased load of ROS-induced cellular damage. Such asynchrony has been proposed for aging mice where the peak in S-phase is delayed (Fig. 3B) and may coincide with maximum ROS levels as evidenced by transcriptional changes consistent with DNA-damage and stress response in IFE stem cells.^54^ Interestingly, calorie restriction, which is linked to longevity, leads to decreased oxidative metabolism in the epidermis.^51^ The link between the clock and aging is further supported by experiments showing that *Bmal1*-mutated mice age prematurely, including in skin,^34,77^ perhaps due to excessive ROS generation.^39,77^

## Concluding Remarks

Stem cell proliferation in epithelial tissues, including the epidermis, is diurnal with the highest proportion of cells in S-phase during the active phase (night in rodents and day in humans). Initial observations of this phenomenon go back 100 years, long before the discovery of the molecular circadian clock mechanism. Subsequent work in the late 20th century characterized these daily cell-cycle oscillations in a variety of epithelial tissues and species at a phenomenological level. But it was not until 10 years ago when it was shown that the intact circadian clock is required for these cell cycle oscillations in epidermal stem cells. Although the circadian clock within keratinocytes is required for these stem cell oscillations, it is not known whether the underlying mechanisms represent direct control of cell cycle regulator expression by the circadian clock or oscillating external signals emanating from keratinocytes and acting on IFE stem cells. Epidermal stem cell proliferation oscillations are dampened when proliferation rate is increased, such as in skin inflammation. Presumably, the circadian clock-mediated dampening in S-phase during the rest phase is suspended when there is an increased need for stem cell proliferation. In vivo studies indicate that the circadian clock coordinates intermediary metabolism and the cell cycle in epidermal stem cells such that glycolysis dominates over oxidative phosphorylation when most cells are in S-phase. This clock-regulated coordination between metabolism and the cell cycle may shield S-phase DNA from damage caused by oxidative metabolism-generated ROS. This hypothesis implies that long-term asynchrony between metabolism and the cell cycle—which can occur during jet lag or when feeding occurs at unusual times of the day—may accelerate epidermal stem cell dysfunction and skin aging.

## Data Availability

No new data were generated or analyzed in support of this research.
